# A Novel ACLY Inhibitor Induces Transcriptional and Metabolic Reprogramming to Overcome Tyrosine kinase inhibitor Resistance in Lymphoma

**DOI:** 10.21203/rs.3.rs-11120406/v1

**Published:** 2026-10-20

**Authors:** Nagarajan Pattabiraman, Cosimo Lobello, David Rushmore, Shengchun Wang, Luca Mologni, Hossein Borghaei, Kavindra Nath, Wujuan Zhang, Aaron R. Goldman, Reza Nejati, Mariusz Wasik, Johnvesly Basappa

## Abstract

Background: Metabolic plasticity contributes to therapeutic resistance in T-cell lymphoma, but the metabolic dependencies underlying tyrosine kinase inhibitor (TKI) resistance remain poorly understood. ATP citrate lyase (ACLY), which converts citrate to cytosolic acetyl-CoA, links glucose metabolism to lipogenesis and acetylation-dependent regulation. We hypothesized that ACLY represents an actionable metabolic vulnerability in lymphoma. Methods: Structure-guided drug discovery and biochemical screening were used to identify the novel ACLY inhibitor VCB1. VCB1 was evaluated against recombinant human ACLY and in TKI-sensitive and TKI-resistant lymphoma models. Antitumor activity was assessed by proliferation, cell-cycle analysis, apoptosis, and immunoblotting, followed by integrated transcriptomic, metabolomic, and lipidomic profiling. Results: Our xenograft study showed that ACLY dependence in ALK+ALCL. We discovered novel ACLY inhibitor VCB1 directly inhibited recombinant ACLY with µM potency (IC₅₀, 0.9 µM) and strongly suppressed lymphoma cell growth. In ALK inhibitor-sensitive SUPM2, VCB1 showed an IC₅₀ of 0.299 µM and retained potency in lorlatinib-resistant SUPM2-LR1000 cells (1.62 µM), whereas BMS-303141 was substantially less active (17.46 and 22.07 µM). VCB1 similarly inhibited BTK inhibitor sensitive and resistant mantle cell lymphoma cell growth (IC₅₀, 0.28 and 0.278 µM), compared with BMS-303141 (30.5 and 37.01 µM). Multi-omics profiling revealed suppression of de novo lipogenesis, including *ACACA, FASN* , and *PPARGC1B* , together with extensive remodeling of triglyceride, phospholipid, cardiolipin, ceramide, and acylcarnitine species and increased expression of fatty-acid oxidation genes. VCB1 activated the integrated stress response (ISR), inducing *ATF4, PPP1R15A, DDIT3, DDIT4* and *SESN2* , while disrupting purine and pyrimidine metabolism and reducing nucleotide biosynthetic genes, including *CAD, DHODH, UCK2* , and *ATIC* . VCB1 further activated an ACLY–ISR–CHAC1 axis associated with reduced GCLC/GCLM expression, glutathione depletion, and a decreased GSH/GSSG ratio, indicating impaired redox homeostasis. TKI-resistant cells exhibited distinct lipid-metabolic remodeling consistent with metabolic plasticity. VCB1 also altered immune-regulatory programs involving *PDCD1, TIGIT, CD247, CD226, NFATC2* , and inflammatory cytokines. Functionally, VCB1 induced cell cycle arrest and apoptosis. Conclusions: ACLY represents an integrated metabolic and signaling vulnerability in lymphoma. VCB1 suppresses lymphoma growth across TKI-sensitive and resistant models by disrupting lipid and nucleotide metabolism, metabolic adaptation and ISR and redox homeostasis supporting ACLY inhibition as a therapeutic strategy for TKI-resistant lymphoma.

